# Disease clearance in ulcerative colitis: Setting the therapeutic goals for future in the treatment of ulcerative colitis

**DOI:** 10.3389/fmed.2022.1102420

**Published:** 2023-01-09

**Authors:** Laura Ramos, Jeny Teo-Loy, Manuel Barreiro-de Acosta

**Affiliations:** ^1^IBD Unit, Department of Gastroenterology, Hospital Universitario de Canarias, Santa Cruz de Tenerife, Spain; ^2^Department of Internal Medicine, University of La Laguna, Santa Cruz de Tenerife, Spain; ^3^IBD Unit, Department of Gastroenterology, University Hospital of Santiago de Compostela, Santiago de Compostela, Spain

**Keywords:** ulcerative colitis, disease clearance, therapeutic goals, outcomes, remission

## Abstract

Ulcerative colitis, one of the phenotypic patterns of inflammatory bowel disease, should be considered a progressive disease with an increased risk of complications if intestinal inflammation is not adequately controlled. The advent of new lines of treatment for this condition has changed and expanded the therapeutic goals to modify its natural history and evolution. The concept of “disease clearance” in ulcerative colitis aims to achieve clinical and biological remission as well as mucosal healing (endoscopic, histological, and in future molecular) in these patients. This review provides the available data on each of the goals of disease clearance in ulcerative colitis to be considered for application in clinical practice in the coming years.

## Introduction

Ulcerative colitis (UC) is one of the phenotypic forms of inflammatory bowel disease (IBD) in which an abnormal inflammation occurs in the colon mucosa and commonly determines diarrhea and the presence of blood in the stool (1). Even the complex mechanisms contributing to the development of UC remain unclear, both innate (neutrophils, dendritic cells, and macrophages) and adaptive immune cells (cytotoxic lymphocytes, regulatory lymphocytes Treg, or helper lymphocytes Th–Th2, Th9, Th17, and Th22) play a crucial role in its pathogenesis influenced by the effect of pro-inflammatory and anti-inflammatory cytokines present in the colonic mucosa (2). Although UC is usually presented as a mild condition (3), over time patients with UC and risk factors for aggressive or complicated disease (4) can develop structural and functional damage to the colon, including colonic dysmotility, benign strictures, and anorectal dysfunction (5). Moreover, a colectomy remains required in up to 20–30% of medically refractory UC (6, 7), and even though the risk of colorectalcarcinoma has decreased over time in patients with UC, it remains elevated in those with associated primary sclerosing cholangitis, long duration of disease, and uncontrolled inflammation (8, 9).

Current pharmacological treatments, including salicylates, thiopurines, small molecules, and biologics, are used in UC according to the severity and extension of the disease, as well as comorbidities and previous response to conventional drugs (10). The principal aim of medical management is to induce and maintain remission with the long-term goals of preventing disease progression (11), including disability, colectomy, and colorectal cancer, and improving the quality of life of UC patients with UC by adequately control of the inflammatory response (7). Out of clinical trials, the recent update of Selecting Therapeutic Targets in Inflammatory Bowel Disease (7) proposes the goals, from short-term to long-term, to monitor treat-to-target strategies in patients with IBD toward altering the natural history of the disease, including clinical indices, biochemical biomarkers, and endoscopic healing, and considering histology healing in UC (7). These goals are incorporated into the concept of “disease clearance (DC),” which represents a deep and complete remission achieving symptomatic remission, mucosal healing (endoscopic and histological healing), and evolving to molecular healing with the restoration of specific molecular pathways involved in the etiopathogenesis of the disease (12). In this review article, we provide updated information from each therapeutic target that could shape the concept of “disease clearance” to improve the long-term outcome of UC.

## Clinical target for disease clearance in UC

Clinical remission is a target in the Selecting Therapeutic Targets in Inflammatory Bowel Disease (STRIDE-2), and it has been ranked as the most important of short-term treatment goals in UC (7). In UC, clinical symptoms are well correlated with the endoscopic (13, 14) and biochemical (15, 16) degree of inflammation and clinical improvement (by the absence of diarrhea and blood in stool) is a predictor of reduced risk of relapse and colectomy (17, 18).

Numerous UC severity indices are available, but the more commonly used in adult clinical trials and studies is the Mayo score (19), which included stool frequency, rectal bleeding, physician’s global assessment, and an endoscopic subscore (ranging from 0 to 12 points). When the endoscopic assessment is excluded, a full clinical index is performed, named the partial Mayo score. Most studies defined clinical remission as a partial Mayo score of ≤ 2 (with no subscore of >1), but this condition allowed the presence of blood in feces (even scarce) and should not be considered as a complete remission. Patient-reported outcome measures (PROMs) are increasingly used to monitor disease activity in clinical practice and as endpoints in clinical trials (20). The PRO2 has become the current standard for assessing symptoms in UC, listed in the STRIDE-2, which included two subjective items of the Mayo score, stool frequency, and rectal bleeding. This PRO2 has been correlated with endoscopic and histological features (21) and allows assessment of clinical remission as a short-term response to treatment in UC. In addition, health-related quality of life (HRQoL) has been suggested as a relevant endpoint for IBD management. IBD impairs both physical and psychological patient’s conditions, and in STRIDE-2, restoration of QoL and absence of disability had been added as a long-term target in UC (7). Several studies have reported that UC impairs QoL and clinical activity (increased bowel frequency, urgency, and rectal bleeding) was pointed out as the factor with the most negative impact on HRQoL (22–24). Moreover, treatments usually used in trials and clinical practice (as aminosalicylates, biologics, and small molecules) are able to improve de QoL in patients with UC (25). The Inflammatory Bowel Disease Questionnaire 32 (IBDQ-32) [a 32-item questionnaire that includes four aspects of the patient’s life and the main domains are intestinal symptoms (10 items), systemic symptoms (five items), and social (12 items) and emotional domains (five items)] and Inflammatory Bowel Disease Questionnaire 36 (IBDQ-36) [a 36-item questionnaire that comprises these points: intestinal symptoms (eight items), systemic symptoms (seven items), social (six items) and emotional domains (eight items), and functional impairment (seven items)] are the most commonly disease-specific tools used and have been demonstrated to be reliable and valid (26).

In summary, clinical remission reaching a normal stool frequency without rectal bleeding is an early goal in patients with UC and attempts a subsequent improvement of the QoL on these patients.

## Endoscopic target for disease clearance in UC

Mucosal healing (MH) is recommended as a therapeutic objective in patients with IBD and included in STRIDE-2 as a long-term treatment goal in UC because it is related to a more favorable course of the disease (7). Even though several endoscopic scores have been used in UC, the Mayo Endoscopic Score (MES) remains the most extensively used endoscopic index in clinical practice and trials (although not validated) because MES is easy and practical, with good predictive value, and provides a simple visual representation of the degree of endoscopic inflammation in UC (from 0 to 3) (27). Another score, Ulcerative Colitis Endoscopic Index of Severity (UCEIS), is also established in STRIDE-2. UCEIS includes three descriptors (vascular pattern, bleeding, and erosions and ulcers) and is particularly accurate in describing the severity of the lesions and treatment responsiveness than MES because considers the size and depth of ulcers. Both, MES and UCEIS, described the most severely affected area of the colon involved but not the extent and/or location of the endoscopic activity (28).

Mucosal healing has been commonly defined as MES of ≤ 1, but complete endoscopic healing represents MES 0 is associated with better disease outcomes, and therefore, differential concepts such as endoscopic improvement (MES of 0 or 1) and endoscopic remission (MES = 0 or UCEIS = 0) should be considered (29). Since 2016, several studies have demonstrated a distinctive outcome according to MES of 0 or 1. In two similarly designed studies, prospective studies (*n* = 187 and 138 patients with UC in clinical remission), a higher percentage of relapse has been observed in patients who had reached an MES 1 index compared with the group with an MES 0 index (36.6% vs. 9.4%; *p* < 0.001 and 19.3 vs. 41%; *p* = 0.022) after a 12-month follow-up (30, 31). Further studies are compilated in a recent meta-analysis reviewing 15 eligible studies, and the rate of clinical relapse for MES 1 patients ranged from 8 to 66.7% and for MES 0 patients from 0 to 33.3%, suggesting that MES = 1 have a higher risk of relapse than a score of MES = 0, which displayed a lower risk of clinical relapse (OR: 0.33; 95% CI: 0.26–0.43; *I*^2^ 13%) irrespective of the follow-up time (12 months or longer). In this meta-analysis, no differences were found comparing MES 0 versus MES 1 regarding the risk of hospitalization or colectomy (32). Moreover, based on a second meta-analysis including 2,608 patients with UC in clinical remission, achieving endoscopic remission (MES 0) had a 52% lower risk of clinical relapse [RR, 0.48 (95% CI: 0.37–0.62)] than those with mild endoscopic activity (MES 1) (33). A recent multicenter study compared between UC patients without disease clearance (DC) (*n* = 385) and patients with UC who reached DC (*n* = 109) (defined as simultaneous clinical (partial Mayo score of ≤ 2), endoscopic (endoscopic Mayo score = 0), and histological (Nancy index = 0) remission). These patients were monitored for more than 12 months, and patients with early disease clearance are at significantly lower risk for hospitalization (5.5% vs. 23.1%; *p* < 0.001) and surgery (1.8% vs. 10.9%; *p* = 0.003) (34), demonstrating a better outcome in these patients when different DC goals are achieved in combination.

In summary, MES 0 should therefore be considered the appropriate therapeutic goal as it predicts a favorable outcome in UC.

## Biological target for disease clearance in UC

Non-invasive serum [as protein C-reactive protein (CRP)] and fecal inflammatory biomarkers [as fecal calprotectin (FC)] are useful for monitoring patients with IBD by regular measures throughout the patient’s disease course. In STRIDE-2, the normalization of CRP and the decrease of FC are short-term and intermediate-term targets in patients with UC, respectively (7). Even though both CRP and FC can predict endoscopic activity (35), a high correlation of FC with clinical, endoscopic, and histological activity has been described (35–37). FC is the most studied biomarker in IBD and involves a cytoplasmatic protein (prominently present in neutrophils) that is released during the inflammatory response in the intestinal mucosa (38). Regarding the FC and its relation with endoscopic mucosal inflammation, a large study (*n* = 115 patients with UC) demonstrates a sensitivity of 93%, specificity of 71%, PPV of 91%, and NPV of 81% using an FC cutoff value of 50 μg/g (39) to identify the inactive disease (non-endoscopic activity), and FC was also able to discriminate inactive from mild, moderate, and highly active disease. In order to predict mucosal healing from inflammation in UC, a study including 75 patients showed that an FC cutoff value of 61 μg/g had sensitivity of 84.1% [75.0–93.2%] and specificity of 83.3% [74.0–92.6%] to differentiate MES = 0 (40). Another study (*n* = 112 UC) determines that the area under the curve (AUC) in receiver operator characteristic analysis of FC to predict Mayo score of 0 and 1 was 0.869 with a cutoff value of 200 μg/g (67% sensitivity and 91% specificity); however, the power of FC to predict Mayo score of 0 was modest because the AUC was 0.639 with a cutoff value of 194 μg/g (71% sensitivity and 58% specificity) (41). A review article to clarify the correlation between FC and histological activity in patients with UC (12 studies and 1,168 patients with UC) shows a clear correlation between FC levels and histology in all included studies; however, 11 different FC calprotectin cutoff points were identified to distinguish histological remission from histological activity, ranging from 40.5 to 250 μg/g (42). In a study of 68 patients with UC, an FC level of ≤ 60 μg/g predicted deeper remission (defined as PRO2 = 0; MES = 0 and Nancy ≤ 1) (area under the curve = 0.91, sensitivity of 83%, and specificity of 90%) (43). Magro et al. (44) evaluated the association between histological scores and the FC levels (*n* = 377 UC) and concluded that the establishment of an FC cutoff value is not as straightforward, with sensitivity and specificity values varying within the same range for thresholds between 150 and 250 μg/g.

In summary, an accurate FC cutoff value for endoscopic and histological remission has not yet been established, but regarding the practical application, an FC of <150 μg/g should be indicative of no inflammation in UC.

## Histological target for disease clearance in UC

In a meta-analysis with 2,265 patients with ulcerative colitis in clinical remission, the benefit of the absence of histological activity as a predictor of clinical remission and preventing the development of complications in the course of the disease have been demonstrated (33). Thus, although there is agreement on the target of achieving endoscopic and clinical remission in patients with ulcerative colitis, histological remission is already an intended long-term goal in STRIDE-2 (7) for these patients. The *ECCO Position Paper: Harmonization of approach to UC Histopathology* has summarized the score systems and the definitions for the assessment of histological features in UC (45). Different scores are available for the assessment of UC inflammation/activity, although the Geboes score (GS) (46) is widely used, only the Robarts histopathology index (RHI) (47) and the Nancy index (NI) (48) have been formally validated. Even when the correlation between the histological scores is good (44, 49), the use of the Nancy index is recommended for observational studies or clinical practice. Histological activity is defined by neutrophil infiltration of epithelium and/or lamina propria; therefore, the minimum requirement for histological remission is the absence of intraepithelial neutrophils, erosions, and ulcerations and corresponds with GS ≤ 2.0, RHI ≤ 3, or NI = 0 (44, 49). Histological remission is superior to endoscopic and clinical remission in predicting clinical outcomes. In endoscopically quiescent UC (MES ≤ 1) (*n* = 66), active histological inflammation (GB > 3.2) was significantly associated with clinical relapse at 18 months (*P* = 0.0005) (44) and shorter time to clinical relapse (*P* = 0.0006) (50). Moreover, complete histological healing (GS = 0) is associated with reduced rates of clinical relapse after 24 months among patients with UC in endoscopic remission (MES ≤ 1) (12% vs. 50%, *p* < 0.001) (51). In a retrospective cohort (*n* = 270), patients with active UC treated-to-target of clinical remission, who achieve and maintain symptomatic remission and endoscopic remission (MES ≤ 1) over consecutive endoscopies (median, 19 months), have a low risk of relapse, particularly in a subset of patients who simultaneously achieve histological remission (NI = 0) (52). Endoscopic and histological evaluation to assess the disease remission should be performed by a complete (pancolonic) colonoscopy as shown in a prospective study (*n* = 325) checking three modes of endoscopic evaluation: “original,” “worst affected,” and “pancolonic” (53). During an extended follow-up (24 months) of UC patients with clearance disease (defined, among other variables, and by histological remission as Nancy = 0) (*n* = 109), a reduction in the risk of hospitalization and surgery (34) is observed. Recently, a case–control study including 45 patients with neoplasia (25 UC) and 353 controls establishes that histological activity (assessed by NI) was associated with an increased risk of colorectal neoplasia (per 1-unit increase, OR: 1.69; 95% CI: 1.29–2.21) (54).

In summary, histological activity in ulcerative colitis *per se* is associated with a worse outcome, thus, these patients should benefit from therapy modifications to obtain prolonged histological remission (GS ≤ 2.0 or NI = 0) and avoid disease progression.

## Molecular target for disease clearance in UC

The transcriptional signature of “inflammation” present in the involved inflamed mucosa of patients with UC had been previously described (55–57) but few studies (58, 59) have been designed to characterize the mucosal signature in “remission or quiescent” colitis. Comparing colonic biopsies from healthy normal controls (total *n* = 29), active colitis (involved inflamed mucosa) (total *n* = 29), and quiescent colitis (involved non-inflamed mucosa) (total *n* = 22), these studies differentiate three patterns: (A) *inflammatory* (gene expression is similar between colitis in remission and mucosa with active inflammation); (B) *healing* (specific to colitis in remission including genes differentially expressed from active colitis and normal control samples); (C) *restoration* to normality (gene expression is similar between colitis in remission and normal control samples) (58, 59). The differentially expressed genes (DEG) differ slightly between both studies due to diverse methodology and no homogeneity in the variables (clinical, endoscopic, and histological) defining quiescent condition but ensuring no flare between 5 and 18 months in each study. Therefore, a compilation of genes is included, and as targets for disease clearance, by controlling the inflammation, improving colonic healing, and approaching mucosa normalization, we will focus on patterns B and C. The genes related to pattern B (healing), a specific transcriptional signature for UC in remission, increased expression of genes involved in O-glycosylation (*MUC17, MUC3A, MUC5AC, MUC12, SPON1*, and *B3GNT3*), several metallopeptidases (MMP1 and MMP3), neutrophil degranulation (CHI3L1), ephrin-mediated repulsion of cells (*EFNB2E, EFNA3, EPHA10*, and *EPHA1*), GAP junction trafficking (*TUBA1C, TUBA4A, TUBB4B, GJB3*, and *CLTB*), and decreased expression of several toll-like receptors (*TLR1, TLR3, TLR5*, and *TLR6*) were observed. The genes expressed in pattern C (restoring to normal) include those that transcribe cell death (NFKSIZ), cellular growth and proliferation (IL-1B and REG1B), cellular migration (IL-1B, IL-8, CXCL5, IL-7R, CXCL-1, and CXCL-3), inflammatory response (DEFB4, IGHR1/4, TOLLIP, SERPINB4, and DEFA5/6), and tissue morphology (MMP10, MMP7, MMP9, and VCAN) (58, 59). Recent studies have defined a molecular signature associated with the remission of ulcerative colitis. Higher expression of ALOX15 (related to eosinophil and mast cells metabolism) was linked to a higher likelihood of remission (56), but an increased risk for a future relapse was associated with higher expression of IL21, IL17F, and IL17A in MES 0/1 patients (60) as well as IL12 and IL23 (61). The molecular profile associated with endoscopic remission (MES ≤ 1) has been linked to increased expression of IFITM1, ITGB2, IL1R2, and IL2RA (62). Even the endoscopic remission (MES ≤ 1) is achieved, minimal inflammation may persist because of histological activity (the presence of neutrophils in the mucosa) and it is related to increased expression of multiple chemokines (CXCL9 and CXCL10), metalloproteinase-encoding genes (MMP7, MMP3, and MMP1), antimicrobial genes (SAA1, SAA2, and LCN2), and genes with a pathogenic role in colorectal carcinogenesis (WTN2, IL17, and DUOXA2) (63, 64).

In summary, the molecular signature of ulcerative colitis in remission continues to present a differential expression compared with healthy controls, which facilitates the development of flares and/or mucosal degeneration. The future design of a “flare predictor molecular profile” could be implemented in clinical practice to modify treatments in a personalized manner.

## Discussion and suggestions

The disease clearance (DC) involves better healing of the inflamed intestinal mucosa in patients with UC. In this review, we have summarized the available data on the features that are required for this healing: clinical, endoscopic, biological, histological, and molecular remission (in future).

**FIGURE 1:**
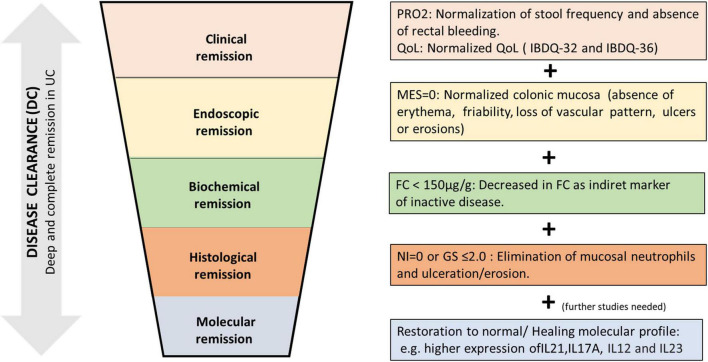
Proposal for disease clearance (DC) in ulcerative colitis (UC). A deep and complete UC remission achieving symptomatic remission, biological remission, mucosal healing (endoscopic and histological remission), and evolving (in future) to molecular healing with the restoration of specific molecular pathways involved in the etiopathogenesis of ulcerative colitis. PRO2, patient’s reported outcome-2; QoL, quality of life; MES, Mayo endoscopic score; FC, fecal calprotectin; NI, Nancy index; GS, Geboes score.

The DC may be achieved when the patient presents normalization of the stool frequency, without traces of blood in stool, with endoscopic remission [without macroscopic inflammation in endoscopy (MES = 0)], reduction of fecal calprotectin (<150 μg/g), histological remission (absence of neutrophil infiltrate in the biopsies of the affected mucosa (GS ≤ 2 or NI = 0), and showing a healing/normalization profile in the molecular study of the biopsies (Figure 1). These rigorous criteria of DC should ensure a better outcome and avoid the progression of UC; therefore, a clinical situation that does not include and combine these goals could not be considered as DC.

Individually, patients that achieve clinical remission or FC normalization or complete mucosal healing will have a better clinical course of the disease, with fewer flares and complications. The study of a combination of all of them is a real challenge in UC, including also histological healing. The STRIDE-2 includes the goals of clinical and endoscopic remission, in addition to the reduction of FC, as short-term and medium-term in the evaluation of response to treatment. However, it still indicates histological remission as desirable, probably because it requires a biopsy (sampling), which limits its incorporation into routine clinical practice.

To achieve better control of intestinal inflammation, avoid relapse and hospital admissions, avoid the need for colectomy, and decrease the risk of CRC, we should try to incorporate this DC condition into the following years, trying to be able to demonstrate that if all these objectives are achieved in a patient, perhaps we could change the natural history of patients with UC.

## Author contributions

LR and MB-d conceived the project. JT-L and LR performed the bibliographic search. LR played a major role in writing the manuscript. MB-d reviewed the manuscript. All authors have approved the final version of the manuscript.

## References

[1] Ungaro R Mehandru S Allen PB Peyrin-Biroulet L Colombel JF. Ulcerative colitis. *Lancet.* (2017) 389:1756–70. 10.1016/S0140-6736(16)32126-2 27914657 PMC6487890

[2] Kałużna A Olczyk P Komosińska-Vassev K. The role of innate and adaptive immune cells in the pathogenesis and development of the inflammatory response in ulcerative colitis. *J Clin Med.* (2022) 11:400. 10.3390/jcm11020400 35054093 PMC8780689

[3] Henriksen M Jahnsen J Lygren I Sauar J Kjellevold Ø Schulz T Ulcerative colitis and clinical course: results of a 5-year population-based follow-up study (the IBSEN study). *Inflamm Bowel Dis.* (2006) 12:543–50. 10.1097/01.MIB.0000225339.91484.fc 16804390

[4] Reinisch W Reinink AR Higgins PD. Factors associated with poor outcomes in adults with newly diagnosed ulcerative colitis. *Clin Gastroenterol Hepatol.* (2015) 13:635–42. 10.1016/j.cgh.2014.03.037 24887059

[5] Torres J Billioud V Sachar DB Peyrin-Biroulet L Colombel JF. Ulcerative colitis as a progressive disease: the forgotten evidence. *Inflammatory bowel diseases.* (2012) 18(7):1356–63. 10.1002/ibd.22839 22162423

[6] Turner D Walsh CM Steinhart AH Griffiths AM. Response to corticosteroids in severe ulcerative colitis: a systematic review of the literature and a meta-regression. *Clin Gastroenterol Hepatol.* (2007) 5:103–10. 10.1016/j.cgh.2006.09.033 17142106

[7] Turner D Ricciuto A Lewis A D’Amico F Dhaliwal J Griffiths AM STRIDE-II: an update on the Selecting Therapeutic Targets in Inflammatory Bowel Disease (STRIDE) Initiative of the International Organization for the Study of IBD (IOIBD): determining therapeutic goals for treat-to-target strategies in IBD. *Gastroenterology.* (2021) 160:1570–83. 10.1053/j.gastro.2020.12.031 33359090

[8] Choi CH Rutter MD Askari A Lee GH Warusavitarne J Moorghen M Forty-year analysis of colonoscopic surveillance program for neoplasia in ulcerative colitis: an updated overview. *Am J Gastroenterol.* (2015) 110:1022–34. 10.1038/ajg.2015.65 25823771 PMC4517513

[9] Beaugerie L Itzkowitz SH. Cancers complicating inflammatory bowel disease. *N Engl J Med.* (2015) 372:1441–52. 10.1056/NEJMra1403718 25853748

[10] Ferretti F Cannatelli R Monico MC Maconi G Ardizzone S. . An update on current pharmacotherapeutic options for the treatment of ulcerative colitis. *J Clin Med.* (2022) 11:2302. 10.3390/jcm11092302 35566428 PMC9104748

[11] Krugliak Cleveland N Torres J Rubin DT. What does disease progression look like in ulcerative colitis, and how might it be prevented? *Gastroenterology.* (2022) 162:1396–408. 10.1053/j.gastro.2022.01.023 35101421

[12] Danese S Roda G Peyrin-Biroulet L. Evolving therapeutic goals in ulcerative colitis: towards disease clearance. *Nat Rev Gastroenterol Hepatol.* (2020) 17:1–2. 31520081 10.1038/s41575-019-0211-1

[13] Turner D Seow CH Greenberg GR Griffiths AM Silverberg MS Steinhart AH. A systematic prospective comparison of noninvasive disease activity indices in ulcerative colitis. *Clin Gastroenterol Hepatol.* (2009) 7:1081–8. 10.1016/j.cgh.2009.06.024 19577010

[14] Restellini S Chao CY Martel M Barkun A Kherad O Seidman E Clinical parameters correlate with endoscopic activity of ulcerative colitis: a systematic review. *Clin Gastroenterol Hepatol.* (2019) 17:1265–75.e8. 10.1016/j.cgh.2018.12.021 30583048

[15] Voiosu T Benguş A Dinu R Voiosu AM Bălănescu P Băicuş C Rapid fecal calprotectin level assessment and the SIBDQ score can accurately detect active mucosal inflammation in IBD patients in clinical remission: a prospective study. *J Gastrointestinal Liver Dis.* (2014) 23:273–8. 10.15403/jgld.2014.1121.233.thv 25267955

[16] Puolanne AM Kolho KL Alfthan H Ristimäki A Mustonen H Färkkilä M. Rapid fecal calprotectin test and symptom index in monitoring the disease activity in colonic inflammatory bowel disease. *Digest Dis Sci.* (2017) 62:3123–30. 10.1007/s10620-017-4770-0 28948412

[17] Turner D Griffiths AM Veerman G Johanns J Damaraju L Blank M Endoscopic and clinical variables that predict sustained remission in children with ulcerative colitis treated with infliximab. *Clin Gastroenterol Hepatol.* (2013) 11:1460–5. 10.1016/j.cgh.2013.04.049 23672831

[18] Arias MT Vande Casteele N Vermeire S de Buck van Overstraeten A Billiet T Baert F A panel to predict long-term outcome of infliximab therapy for patients with ulcerative colitis. *Clin Gastroenterol Hepatol.* (2015) 13:531–8. 10.1016/j.cgh.2014.07.055 25117777

[19] Schroeder KW Tremaine WJ Ilstrup DM. Coated oral 5-aminosalicylic acid therapy for mildly to moderately active ulcerative colitis. A randomized study. *N Engl J Med.* (1987) 317:1625–9. 10.1056/NEJM198712243172603 3317057

[20] de Jong MJ Huibregtse R Masclee AAM Jonkers D Pierik MJ. Patient-reported outcome measures for use in clinical trials and clinical practice in inflammatory bowel diseases: a systematic review. *Clin Gastroenterol Hepatol.* (2018) 16:648–63.e3. 10.1016/j.cgh.2017.10.019 29074448

[21] Dragasevic S Sokic-Milutinovic A Stojkovic Lalosevic M Milovanovic T Djuranovic S Jovanovic I Correlation of Patient-Reported Outcome (PRO-2) with endoscopic and histological features in ulcerative colitis and crohn’s disease patients. *Gastroenterol Res Pract.* (2020) 2020:2065383. 10.1155/2020/2065383 32328091 PMC7154964

[22] Verma S Tsai HH Giaffer MH. Does better disease-related education improve quality of life? A survey of IBD patients. *Digest Dis Sci.* (2001) 46:865–9. 10.1023/A:1010725106411 11330426

[23] Hoivik ML Moum B Solberg IC Cvancarova M Hoie O Vatn MH Health-related quality of life in patients with ulcerative colitis after a 10-year disease course: results from the IBSEN study. *Inflamm Bowel Dis.* (2012) 18:1540–9. 10.1002/ibd.21863 21936030

[24] Han SW McColl E Barton JR James P Steen IN Welfare MR. Predictors of quality of life in ulcerative colitis: the importance of symptoms and illness representations. *Inflamm Bowel Dis.* (2005) 11:24–34. 10.1097/00054725-200501000-00004 15674110

[25] Calviño-Suárez C Ferreiro-Iglesias R Bastón-Rey I Barreiro-de Acosta M. Role of quality of life as endpoint for inflammatory bowel disease treatment. *Int J Environ Res Public Health.* (2021) 18:7159. 10.3390/ijerph18137159 34281095 PMC8296948

[26] Yarlas A Maher S Bayliss M Lovley A Cappelleri JC Bushmakin AG The inflammatory bowel disease questionnaire in randomized controlled trials of treatment for ulcerative colitis: systematic review and meta-analysis. *J Patient Center Res Rev.* (2020) 7:189–205. 10.17294/2330-0698.1722 32377552 PMC7197888

[27] Lee JS Kim ES Moon W. Chronological review of endoscopic indices in inflammatory bowel disease. *Clin Endosc.* (2019) 52:129–36. 30130840 10.5946/ce.2018.042 PMC6453843

[28] Ikeya K Hanai H Sugimoto K Osawa S Kawasaki S Iida T The ulcerative colitis endoscopic index of severity more accurately reflects clinical outcomes and long-term prognosis than the mayo endoscopic score. *J Crohns Colitis.* (2016) 10:286–95. 10.1093/ecco-jcc/jjv210 26581895 PMC4957474

[29] Sharara AI Malaeb M Lenfant M Ferrante M. Assessment of endoscopic disease activity in ulcerative colitis: is simplicity the ultimate sophistication? *Inflamm Intestinal Dis.* (2022) 7:7–12. 10.1159/000518131 35224012 PMC8820167

[30] Barreiro-de Acosta M Vallejo N de la Iglesia D Uribarri L Bastón I Ferreiro-Iglesias R Evaluation of the risk of relapse in ulcerative colitis according to the degree of mucosal healing (Mayo 0 vs 1): a longitudinal cohort study. *J Crohn’s Colitis.* (2016) 10:13–9. 10.1093/ecco-jcc/jjv158 26351390

[31] Boal Carvalho P Dias de Castro F Rosa B Moreira MJ Cotter J. Mucosal healing in ulcerative colitis–when zero is better. *J Crohn’s Colitis.* (2016) 10:20–5. 10.1093/ecco-jcc/jjv180 26438714

[32] Viscido A Valvano M Stefanelli G Capannolo A Castellini C Onori E Systematic review and meta-analysis: the advantage of endoscopic Mayo score 0 over 1 in patients with ulcerative colitis. *BMC Gastroenterol.* (2022) 22:92. 10.1186/s12876-022-02157-5 35240984 PMC8895505

[33] Yoon H Jangi S Dulai PS Boland BS Prokop LJ Jairath V Incremental benefit of achieving endoscopic and histologic remission in patients with ulcerative colitis: a systematic review and meta-analysis. *Gastroenterology.* (2020) 159:1262–75.e7. 10.1053/j.gastro.2020.06.043 32585306 PMC7658293

[34] D’Amico F Fiorino G Solitano V Massarini E Guillo L Allocca M Ulcerative colitis: impact of early disease clearance on long-term outcomes - A multicenter cohort study. *U Eur Gastroenterol J.* (2022) 10:775–82. 10.1002/ueg2.12288 36107109 PMC9486490

[35] Sands BE. Biomarkers of inflammation in inflammatory bowel disease. *Gastroenterology.* (2015) 149:1275–85.e2. 10.1053/j.gastro.2015.07.003 26166315

[36] Theede K Holck S Ibsen P Kallemose T Nordgaard-Lassen I Nielsen AM. Fecal calprotectin predicts relapse and histological mucosal healing in ulcerative colitis. *Inflamm Bowel Dis.* (2016) 22:1042–8. 10.1097/MIB.0000000000000736 26919460

[37] Tibble JA Sigthorsson G Bridger S Fagerhol MK Bjarnason I. Surrogate markers of intestinal inflammation are predictive of relapse in patients with inflammatory bowel disease. *Gastroenterology.* (2000) 119:15–22. 10.1053/gast.2000.8523 10889150

[38] Røseth AG Aadland E Jahnsen J Raknerud N. Assessment of disease activity in ulcerative colitis by faecal calprotectin, a novel granulocyte marker protein. *Digestion.* (1997) 58:176–80. 10.1159/000201441 9144308

[39] Schoepfer AM Beglinger C Straumann A Trummler M Renzulli P Seibold F. Ulcerative colitis: correlation of the Rachmilewitz endoscopic activity index with fecal calprotectin, clinical activity, C-reactive protein, and blood leukocytes. *Inflamm Bowel Dis.* (2009) 15:1851–8. 10.1002/ibd.20986 19462421

[40] Kristensen V Klepp P Cvancarova M Røseth A Skar V Moum B. Prediction of endoscopic disease activity in ulcerative colitis by two different assays for fecal calprotectin. *J Crohn’s Colitis.* (2015) 9:164–9. 10.1093/ecco-jcc/jju015 25518057

[41] Yamaguchi S Takeuchi Y Arai K Fukuda K Kuroki Y Asonuma K Fecal calprotectin is a clinically relevant biomarker of mucosal healing in patients with quiescent ulcerative colitis. *J Gastroenterol Hepatol.* (2016) 31:93–8. 10.1111/jgh.13061 26212346

[42] D’Amico F Bonovas S Danese S Peyrin-Biroulet L. Review article: faecal calprotectin and histologic remission in ulcerative colitis. *Aliment Pharmacol Therapeut.* (2020) 51:689–98. 10.1111/apt.15662 32048751

[43] Patel A Panchal H Dubinsky MC. Fecal calprotectin levels predict histological healing in ulcerative colitis. *Inflamm Bowel Dis.* (2017) 23:1600–4. 10.1097/MIB.0000000000001157 28590341

[44] Magro F Lopes J Borralho P Lopes S Coelho R Cotter J Comparison of different histological indexes in the assessment of UC activity and their accuracy regarding endoscopic outcomes and faecal calprotectin levels. *Gut.* (2019) 68:594–603. 10.1136/gutjnl-2017-315545 29437913

[45] Magro F Doherty G Peyrin-Biroulet L Svrcek M Borralho P Walsh A ECCO position paper: harmonization of the approach to ulcerative colitis histopathology. *J Crohn’s Colitis.* (2020) 14:1503–11. 10.1093/ecco-jcc/jjaa110 32504534

[46] Geboes K Riddell R Ost A Jensfelt B Persson T Löfberg R. A reproducible grading scale for histological assessment of inflammation in ulcerative colitis. *Gut.* (2000) 47:404–9. 10.1136/gut.47.3.404 10940279 PMC1728046

[47] Mosli MH Feagan BG Zou G Sandborn WJ D’Haens G Khanna R Development and validation of a histological index for UC. *Gut.* (2017) 66:50–8. 26475633 10.1136/gutjnl-2015-310393

[48] Marchal-Bressenot A Salleron J Boulagnon-Rombi C Bastien C Cahn V Cadiot G Development and validation of the Nancy histological index for UC. *Gut.* (2017) 66:43–9. 10.1136/gutjnl-2015-310187 26464414

[49] Magro F Lopes J Borralho P Lopes S Coelho R Cotter J Comparing the continuous geboes score with the robarts histopathology index: definitions of histological remission and response and their relation to faecal calprotectin levels. *J Crohn’s Colitis.* (2020) 14:169–75. 10.1093/ecco-jcc/jjz123 31504348

[50] Kevans D Kirsch R Dargavel C Kabakchiev B Riddell R Silverberg MS. Histological markers of clinical relapse in endoscopically quiescent ulcerative colitis. *Inflamm Bowel Dis.* (2020) 26:1722–9. 10.1093/ibd/izz308 31883337 PMC8243631

[51] Cushing KC Tan W Alpers DH Deshpande V Ananthakrishnan AN. Complete histologic normalisation is associated with reduced risk of relapse among patients with ulcerative colitis in complete endoscopic remission. *Aliment Pharmacol Therapeut.* (2020) 51:347–55. 10.1111/apt.15568 31696961 PMC6980269

[52] Jangi S Holmer AK Dulai PS Boland BS Collins AE Pham L Risk of relapse in patients with ulcerative colitis with persistent endoscopic healing: a durable treatment endpoint. *J Crohn’s Colitis.* (2021) 15:567–74. 32914194 10.1093/ecco-jcc/jjaa184 PMC8023862

[53] Kaneshiro M Takenaka K Suzuki K Fujii T Hibiya S Kawamoto A Pancolonic endoscopic and histologic evaluation for relapse prediction in patients with ulcerative colitis in clinical remission. *Aliment Pharmacol Therapeut.* (2021) 53:900–7. 10.1111/apt.16310 33645736

[54] Kirchgesner J Svrcek M Le Gall G Landman C Dray X Bourrier A Nancy index scores of chronic inflammatory bowel disease activity associate with development of colorectal neoplasia. *Clin Gastroenterol Hepatol.* (2020) 18:150–7.e1. 10.1016/j.cgh.2019.05.002 31085339

[55] Linggi B Jairath V Zou G Shackelton LM McGovern DPB Salas A Meta-analysis of gene expression disease signatures in colonic biopsy tissue from patients with ulcerative colitis. *Sci Rep.* (2021) 11:18243. 10.1038/s41598-021-97366-5 34521888 PMC8440637

[56] Haberman Y Karns R Dexheimer PJ Schirmer M Somekh J Jurickova I Ulcerative colitis mucosal transcriptomes reveal mitochondriopathy and personalized mechanisms underlying disease severity and treatment response. *Nat Communicat.* (2019) 10:38. 10.1038/s41467-018-07841-3 PMC6318335 30604764

[57] Taman H Fenton CG Hensel IV Anderssen E Florholmen J Paulssen RH. Transcriptomic landscape of treatment-naïve ulcerative colitis. *J Crohn’s Colitis.* (2018) 12:327–36. 10.1093/ecco-jcc/jjx139 29040430 PMC6290885

[58] Planell N Lozano JJ Mora-Buch R Masamunt MC Jimeno M Ordás I Transcriptional analysis of the intestinal mucosa of patients with ulcerative colitis in remission reveals lasting epithelial cell alterations. *Gut.* (2013) 62:967–76. 10.1136/gutjnl-2012-303333 23135761

[59] Fenton CG Taman H Florholmen J Sørbye SW Paulssen RH. Transcriptional signatures that define ulcerative colitis in remission. *Inflamm Bowel Dis.* (2021) 27:94–105. 10.1093/ibd/izaa075 32322884 PMC7737162

[60] Fukaura K Iboshi Y Ogino H Ihara E Nakamura K Nishihara Y Mucosal profiles of immune molecules related to T helper and regulatory t cells predict future relapse in patients with quiescent ulcerative colitis. *Inflamm Bowel Dis.* (2019) 25:1019–27. 10.1093/ibd/izy395 30668727

[61] Uchiyama K Takagi T Mizushima K Kajiwara-Kubota M Kashiwagi S Toyokawa Y Increased mucosal IL-12 expression is associated with relapse of ulcerative colitis. *BMC Gastroenterol.* (2021) 21:122. 33730998 10.1186/s12876-021-01709-5 PMC7968323

[62] Román J Planell N Lozano JJ Aceituno M Esteller M Pontes C Evaluation of responsive gene expression as a sensitive and specific biomarker in patients with ulcerative colitis. *Inflamm Bowel Dis.* (2013) 19:221–9. 22605655 10.1002/ibd.23020

[63] Hernández-Rocha C Nayeri S Turpin W Steel M Borowski K Stempak JM Combined histo-endoscopic remission but not endoscopic healing alone in ulcerative colitis is associated with a mucosal transcriptional profile resembling healthy mucosa. *J Crohn’s Colitis.* (2022) 16:1020–9. 10.1093/ecco-jcc/jjac001 34999763 PMC9351979

[64] Manuc M Ionescu EM Milanesi E Dobre M Tieranu I Manuc TE Molecular signature of persistent histological inflammation in ulcerative colitis with mucosal healing. *J Gastrointestin Liver Dis.* (2020) 29:159–66. 32530982 10.15403/jgld-576

